# Supplementary material for: An osteoporosis course as a separate component of problem-based learning
Source: PLoS One. 2025 Nov 14;20(11):e0336915. doi: 10.1371/journal.pone.0336915 (PMC12617839; doi:10.1371/journal.pone.0336915)
Supplement: S1 Graph — (DOCX) [file pone.0336915.s001.docx]

Number of points acquired

-19

-14

-9

-4

1

6

11

16

21

Control Group I

Study Group I

70% threshold for the written test

**S1 Graph**.

Comparison of points acquired by Study Group I (students who completed the Elective Osteoporosis Course I) and Control Group I (students who completed only the mandatory curriculum). The x-axis shows the distribution of total test scores, and the y-axis represents the number of students in each category. The dashed vertical line indicates the 70% threshold required to pass the written test. Boxplots illustrate the distribution of scores: the box shows the interquartile range (IQR) - from the 25th percentile (Q1, lower edge) to the 75th percentile (Q3, upper edge); the horizontal line inside each box marks the median (50th percentile); the whiskers (thin lines with caps) extend to the most extreme values within 1.5 × IQR from Q1 and Q3 (the “adjacent values”); and individual dots represent outliers beyond this range.
