# Supplementary figures and images for: An osteoporosis course as a separate component of problem-based learning
Source: PLoS One. 2025 Nov 14;20(11):e0336915. doi: 10.1371/journal.pone.0336915 (PMC12617839; doi:10.1371/journal.pone.0336915)

**Data for Graph 1**


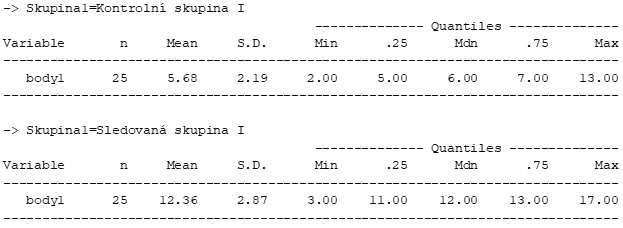


**Data for Graph 2**


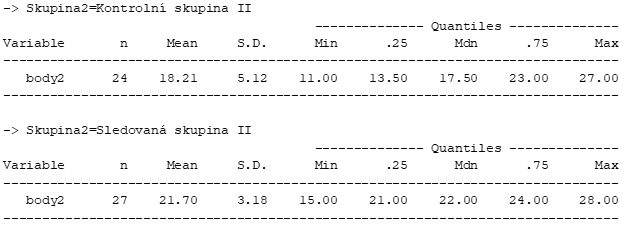

Supplement: S1 Data — (DOCX) [file pone.0336915.s003.docx]
