# Supplementary material for: An osteoporosis course as a separate component of problem-based learning
Source: PLoS One. 2025 Nov 14;20(11):e0336915. doi: 10.1371/journal.pone.0336915 (PMC12617839; doi:10.1371/journal.pone.0336915)
Supplement: Attachment — Submitted filename: comments.pdf [file pone.0336915.s006.pdf]

PLOS ONE

An Osteoporosis Course as a Separate Component of Problem-Based Learning

--Manuscript Draft--

**Manuscript Number:**	PONE-D-25-15083
**Article Type:**	Research Article
**Full Title:**	An Osteoporosis Course as a Separate Component of Problem-Based Learning
**Short Title:**	An Elective Osteoporosis Course
**Corresponding Author:**	Iva Hoffmanová, Ph.D.<br>Charles University Second Faculty of Medicine: Univerzita Karlova 2 lekarska fakulta<br>Prague 5, CZECHIA
**Keywords:**	problem-based learning, principles of clinical medicine, osteoporosis
**Abstract:**	Introduction: The objective was to compare the extent of acquired knowledge regarding osteoporosis-related issues in a group of medical students who successfully completed an Elective Course on Osteoporosis as part of problem-based learning, and a group of medical students who completed only the mandatory curriculum.<br>Study groups and methods: Study group I comprised of 25 fourth-year students who successfully completed the Elective Osteoporosis Course I (focused on pathophysiology and diagnostics), while control group I comprised of 25 fifth-year students who successfully completed all required fourth-year courses, but did not participate in the elective Osteoporosis I course. Study group II comprised of 27 fourth-year students who successfully completed the Elective Osteoporosis Course II (focused on treatment of osteoporotic fractures), while control group II comprised of 24 sixth-year students who were preparing for final exams in surgical disciplines, but did not participated in the Elective Osteoporosis Course II. The groups were compared using a linear regression model with robust estimation of standard errors using Stata 13.1. A p-value < 0.05 was considered statistically significant.<br>Results: Study group I scored in multiple-choice testing, on average, 6.7 points higher than control group I. Study group II scored in multiple-choice testing, on average, 3.5 points higher than control group II. Both differences was statistically significant (p < 0.001, R2 = 0.64, 95% CI: 5.2-8.1; p = 0.006, R2 = 0.15, 95% CI: 1.1-5.9; respectively).<br>Conclusion: Results demonstrated a greater knowledge in students who participated in problem-based learning medical studies relative to those who completed only the mandatory curriculum.
**Order of Authors:**	Iva Hoffmanová, Ph.D.
	Valér Džupa, prof. Ph.D.
	Petr Waldauf, Ph.D.
	Robert Grill, Ph.D.
	Václav Báča, prof. Ph.D.
**Additional Information:**	
**Question**	**Response**
**Financial Disclosure**<br><br>Enter a financial disclosure statement that describes the sources of funding for the work included in this submission. Review the submission guidelines for detailed requirements. View published research articles from *PLOS ONE* for specific examples.	The author(s) received no specific funding for this work.

This statement is required for submission and **will appear in the published article** if the submission is accepted. Please make sure it is accurate.<br><br>**Funded studies**<br>Enter a statement with the following details:<br>• Initials of the authors who received each award<br>• Grant numbers awarded to each author<br>• The full name of each funder<br>• URL of each funder website<br>• Did the sponsors or funders play any role in the study design, data collection and analysis, decision to publish, or preparation of the manuscript?<br><br>Did you receive funding for this work?	
**Competing Interests**<br><br>Use the instructions below to enter a competing interest statement for this submission. On behalf of all authors, disclose any competing interests that could be perceived to bias this work—acknowledging all financial support and any other relevant financial or non-financial competing interests.<br><br>This statement is required for submission and **will appear in the published article** if the submission is accepted. Please make sure it is accurate and that any funding sources listed in your Funding Information later in the submission form are also declared in your Financial Disclosure statement.<br><br>View published research articles from *PLOS ONE* for specific examples.	NO authors have competing interests

**NO authors have competing interests**<br><br>Enter: *The authors have declared that no competing interests exist.*<br><br>**Authors with competing interests**<br><br>Enter competing interest details beginning with this statement:<br><br>*I have read the journal's policy and the authors of this manuscript have the following competing interests: [insert competing interests here]*<br><br>* typeset	
**Ethics Statement**<br><br>Enter an ethics statement for this submission. This statement is required if the study involved:<br><br>• Human participants<br>• Human specimens or tissue<br>• Vertebrate animals or cephalopods<br>• Vertebrate embryos or tissues<br>• Field research<br><br>Write "N/A" if the submission does not require an ethics statement.<br><br>General guidance is provided below. Consult the submission guidelines for detailed instructions. **Make sure that all information entered here is included in the Methods section of the manuscript.**	Our study does not involve human subjects. It focuses on evaluating knowledge outcomes under different pedagogical approaches in teaching medical students about osteoporosis

Powered by Editorial Manager® and ProduXion Manager® from Aries Systems Corporation

**Format for specific study types**<br><br>**Human Subject Research (involving human participants and/or tissue)**<br>• Give the name of the institutional review board or ethics committee that approved the study<br>• Include the approval number and/or a statement indicating approval of this research<br>• Indicate the form of consent obtained (written/oral) or the reason that consent was not obtained (e.g. the data were analyzed anonymously)<br><br>**Animal Research (involving vertebrate animals, embryos or tissues)**<br>• Provide the name of the Institutional Animal Care and Use Committee (IACUC) or other relevant ethics board that reviewed the study protocol, and indicate whether they approved this research or granted a formal waiver of ethical approval<br>• Include an approval number if one was obtained<br>• If the study involved *non-human primates*, add *additional details* about animal welfare and steps taken to ameliorate suffering<br>• If anesthesia, euthanasia, or any kind of animal sacrifice is part of the study, include briefly which substances and/or methods were applied<br><br>**Field Research**<br><br>Include the following details if this study involves the collection of plant, animal, or other materials from a natural setting:<br>• Field permit number<br>• Name of the institution or relevant body that granted permission	
**Data Availability**<br><br>Authors are required to make all data underlying the findings described fully available, without restriction, and from the time of publication. PLOS allows rare exceptions to address legal and ethical concerns. See the PLOS Data Policy and FAQ for detailed information.	Yes - all data are fully available without restriction

A Data Availability Statement describing where the data can be found is required at submission. Your answers to this question constitute the Data Availability Statement and **will be published in the article**, if accepted.<br><br>**Important:** Stating 'data available on request from the author' is not sufficient. If your data are only available upon request, select 'No' for the first question and explain your exceptional situation in the text box.<br><br>Do the authors confirm that all data underlying the findings described in their manuscript are fully available without restriction?	
**Describe where the data may be found in full sentences. If you are copying our sample text, replace any instances of XXX with the appropriate details.**<br><br>• If the data are **held or will be held in a public repository**, include URLs, accession numbers or DOIs. If this information will only be available after acceptance, indicate this by ticking the box below. For example: *All XXX files are available from the XXX database (accession number(s) XXX, XXX.*).<br>• If the data are all contained **within the manuscript and/or Supporting Information files**, enter the following: *All relevant data are within the manuscript and its Supporting Information files.*<br>• If neither of these applies but you are able to provide **details of access elsewhere**, with or without limitations, please do so. For example:<br><br>*Data cannot be shared publicly because of [XXX]. Data are available from the XXX Institutional Data Access / Ethics Committee (contact via XXX) for researchers who meet the criteria for access to confidential data.*<br><br>*The data underlying the results presented in the study are available from (include the name of the third party*	The data are available in the repository of the Third Faculty of Medicine, Charles University.

and contact information or URL).<br>• This text is appropriate if the data are owned by a third party and authors do not have permission to share the data.<br><br>* typeset	
Additional data availability information:	

Manuscript
Click here to access/download;Manuscript;Osteoporosis_manuscript.docx

Title: An Osteoporosis Course as a Separate Component of Problem-Based Learning

Short version of the title: An Elective Osteoporosis Course

Iva Hoffmanová, MD, PhD[1]; Valér Džupa, MD, PhD, Prof[2]; Petr Waldauf, MD, PhD[3], Robert Grill, MD, PhD[4], Václav Báča, MD, PhD, Prof[5].

[1]Department of Internal Medicine, Second Faculty of Medicine, Charles University, Prague, Czech Republic

[2]Department of Orthopedics and Traumatology, Third Faculty of Medicine, Charles University, Prague, Czech Republic

[3]Department of Anesthesiology and Resuscitation, Third Faculty of Medicine, Charles University, Prague, Czech Republic

[4]Department of Urology, Third Faculty of Medicine, Charles University, Prague, Czech Republic

[5]Department of Anatomy, Third Faculty of Medicine, Charles University, Prague, Czech Republic

Corresponding autor:
Iva Hoffmanová, M.D., Ph.D.
Department of Internal Medicine
Second Faculty of Medicine, Charles University
V Úvalu 84, 150 06 Prague 5,
Czech Republic
Phone: +420 723705493
Fax number: +420 224434019
E-mail address: iva.hoffmanova@gmail.com

No conflicts of interest and no source of funding have been stated by all authors.

Abstract

Introduction: The objective was to compare the extent of acquired knowledge regarding osteoporosis-related issues in a group of medical students who successfully completed an Elective Course on Osteoporosis as part of problem-based learning, and a group of medical students who completed only the mandatory curriculum.

Study groups and methods: Study group I comprised of 25 fourth-year students who successfully completed the Elective Osteoporosis Course I (focused on pathophysiology and diagnostics), while control group I comprised of 25 fifth-year students who successfully completed all required fourth-year courses, but did not participate in the elective Osteoporosis I course. Study group II comprised of 27 fourth-year students who successfully completed the Elective Osteoporosis Course II (focused on treatment of osteoporotic fractures), while control group II comprised of 24 sixth-year students who were preparing for final exams in surgical disciplines, but did not participated in the Elective Osteoporosis Course II. The groups were compared using a linear regression model with robust estimation of standard errors using Stata 13.1. A p-value < 0.05 was considered statistically significant.

Results: Study group I scored in multiple-choice testing, on average, 6.7 points higher than control group I. Study group II scored in multiple-choice testing, on average, 3.5 points higher than control group II. Both differences was statistically significant ($p < 0.001$, $R^2 = 0.64$, 95% CI: 5.2-8.1; $p = 0.006$, $R^2 = 0.15$, 95% CI: 1.1-5.9; respectively).

Conclusion: Results demonstrated a greater knowledge in students who participated in problem-based learning medical studies relative to those who completed only the mandatory curriculum.

Keywords: problem-based learning, principles of clinical medicine, osteoporosis

Introduction

Osteoporosis is an emerging healthcare issue. Therefore, we included Elective Osteoporosis Course to the medical teaching at our Faculty of Medicine. Problem-based learning (PBL) in pre-clinical medical studies is considered the optimal information integration method for theoretical and clinical disciplines (1–3). However, it appears that elective courses on topics studied across multiple disciplines (as a component of basic clinical problems addressed in several mandatory courses) can also serve as a suitable form of PBL supplementation (4–6). Osteoporosis is considered to be one such interdisciplinary topic (7–11). In the context of PBL, the subject of osteoporosis is incorporated into five mandatory courses at our faculty: 1/ Diagnostics based on imaging techniques; 2/ Hematology and Oncology; 3/ Diabetology, Endocrinology, Gastroenterology, and Abdominal Surgery; 4/ Nephrology, Urology, Rheumatology and Geriatrics, and 5/ Orthopedics, Traumatology, Anesthesiology and Resuscitation. We prepared an Elective Osteoporosis Course in an effort to consolidate the osteoporosis-related information presented in the above-named mandatory courses. The aim of this study was to compare the depth of acquired knowledge concerning osteoporosis-related issues in two groups of students who successfully completed the Elective Osteoporosis Course, and two groups of students who did not participate in the course.

Materials and methods

The Elective Osteoporosis Course was divided into two sub-courses. Elective Osteoporosis Course I focused on the definition, clinical picture, biomechanics, and metabolism of bone, as well as biochemical, densitometric and other methods used in the diagnosis of osteoporosis. Elective Osteoporosis Course II was dedicated to the disease course and treatment of typical osteoporotic fractures. Each sub-course consisted of 14 study hours comprised from 10 hours of self-study with e-learning materials (http://osteokurz.lf3.cuni.cz/) and two interactive seminars, each 2 hours in length, intended to augment knowledge acquired during self-study with analyses of illustrative case studies. To receive credit for the course, students had to receive a minimum score of 70% on the final test. The test was composed of 10 multiple-choice questions with answers of various weight, selected from two pools of 100 questions that had been prepared for each of the two courses. These questions were a component of the e-learning materials in the form of text and a demo version of the test (i.e., it was possible to attempt a test composed of randomly selected questions with immediate on-line results, without prior-known values of individual responses; it was also possible to retake the test, with each reattempt consisting of a different set of 10 questions randomly selected from the pool of 100).

The study comparisons always involved two groups of students. Study group I comprised 25 fourth-year students who successfully completed the Elective Osteoporosis Course I. Control group I comprised 25 fifth-year students who successfully completed all required fourth-year courses pertaining to osteoporosis and related issues, but did not participate in the Elective Osteoporosis Course I. Study group II comprised 27 fourth-year students who successfully completed the Elective Osteoporosis Course II. Control group II consisted of 24 sixth-year students who did not participate in the Elective Osteoporosis Course II, but successfully completed all required fourth-year courses pertaining to osteoporosis and related issues, as well

as fifth-year courses in Orthopedics and Traumatology of the musculoskeletal system, and were preparing for final exams in surgical disciplines at the time of testing.

The groups were compared using a linear regression model with robust estimation of standard errors using Stata 13.1. A p-value < 0.05 was considered statistically significant. The results are presented as linear regression coefficients (difference between means) with 95% confidence intervals and coefficient of determination (R^2).

Results

Elective Osteoporosis Course I Test Results: The number of points acquired on the test ranged from −16 to +21 points. A 70% threshold was set for successfully passing the test; it was fixed at 10 points (approximately 5 points more than students could achieve in the event they marked all choices in all answers). Point gains are presented in Table 1, which indicates that 24 (96%) students from study group I, and 1 (4%) student from control group I, successfully passed the test. An overview of the point distribution is presented in Table 1. Graph 1 shows that study group I achieved an average of 6.7 points more than control group I, and this difference was significant ($p < 0.001$, $R^2 = 0.64$, 95% CI: 5.2-8.1).

Elective Osteoporosis Course II Test Results: The number of points acquired on the test ranged from −21 to +28 points. A 70% threshold was set for successfully passing the test; it was fixed at 14 points (approximately 7 points more than students could achieve in the event they marked choices in all all answers). Twenty-seven (100%) of students from study group II, and 17 (71%) students from control group II, successfully passed the test. An overview of the point distribution is presented in Table 2. Graph 2 demonstrates that study group II achieved an

average of 3.5 points more than control group II and this difference was significant ($p = 0.006$, $R^2 = 0.15$, 95% CI: 1.1-5.9).

Discussion

We created an Elective Osteoporosis Course for third and fourth-year medical students in the form of an individual and integrated Problem-Based Learning Course. It was driven by the conviction that osteoporosis is a serious issue in contemporary medicine, which deserves special attention in the undergraduate study of general medicine.

In the Elective Osteoporosis Course, we selected a combination of e-learning studies and case study analyses via interactive seminars for three reasons: 1/ contemporary medical students at our faculty prefer e-learning materials, 2/ case studies best depict the contemporary concept of education in terms of Case-Based Learning (12–15), 3/ the authors of this article have had positive experiences with e-learning studies used by junior doctors preparing for specialized exams (Educational Center for Anatomy and Endoscopy: http://ecae.lf3.cuni.cz/ and the Center for Integrated Study of the Pelvis: http://medical-cisp.lf3.cuni.cz/). It turned out that osteoporosis was a suitable choice for creating an elective course, as more than 250 students have completed the course over its 12 years of existence.

The considerable difference observed in the results of study group I and control group I can be ascribed to the fact that the Elective Osteoporosis Course I was a theoretically oriented course. Fifth-year medical students are more interested in the clinical problems of individual nosological units and therefore retain less information concerning etiology, pathogenesis and laboratory diagnostics of osteoporosis in comparison to information concerning the clinical picture of individual consequences of the disease. This may also explains why the differences observed in

the results of study group II and control group II were less pronounced. The sixth-year students had already completed courses in Orthopedics and Traumatology of the musculoskeletal system, during which individual osteoporotic fractures are examined via interactive seminars and analyses of typical case studies.

A number of studies concerning the efficacy of teaching medicine in the form of a PBL curriculum have already been conducted (16–19, 20, 21). Nevertheless, we believe that the results of our study have shown that the organization of elective, supplementary topic-based courses taught via PBL (and in various courses) is the correct path for long-term retention of student knowledge.

As other authors have previously expressed (12–15), we consider the incorporation of case studies in PBL, and their detailed critical analyses during instruction, to be very important. We believe that control group II demonstrated substantially better understanding in comparison to control group I simply because, during the fifth and sixth years of medical school, the study of surgical disciplines (in the context of clinical preparation) is, to a large extent, based on critical reviews of case reports.

Conclusion

This study has unequivocally proved a higher degree of knowledge in students of problem-based medical studies who, in addition to completing the standard osteoporosis curriculum in mandatory courses, participated in a specialized, elective e-learning course focused on the revision and expansion of osteoporosis-related information across various disciplines. Furthermore, the study also supports the well-known thesis regarding the suitability of

incorporating case studies into the teaching of preclinical and clinical disciplines, which enables better retention of information acquired during theoretical instruction.

Lessons for Practice

Problem-based learning is very efficient means for communicating the latest disease-related information across multiple medical disciplines. This is especially true for diseases such as osteoporosis, which is an increasingly pressing issue in medicine. To effectively treat osteoporosis, healthcare professionals need a thorough understanding of the latest advances in treatment as well as the latest information regarding pathophysiology. Both topics are well suited for the problem-based learning approach to continuing medical education.

References

1. Gwee MC. Problem-based learning: a strategic learning system design for the education of healthcare professionals in the 21st century. The Kaohsiung journal of medical sciences 2009; 25(5):231–9.

2. Onyon C. Problem-based learning: a review of the educational and psychological theory. The clinical teacher 2012; 9(1):22–6.

3. Worm BS. Learning from simple ebooks, online cases or classroom teaching when acquiring complex knowledge. A randomized controlled trial in respiratory physiology and pulmonology. PloS one 2013; 8(9):e73336.

4. Bergelt C, Lauke H, Petersen-Ewert C, Jucker M, Bauer CK. "Alcohol and nicotine"--Concept and evaluation of an interdisciplinary elective course with OSPE in preclinical medical education. GMS Zeitschrift fur medizinische Ausbildung 2014; 31(1):Doc9.

5. Puljak L, Sapunar D. Web-based elective courses for medical students: an example in pain. Pain medicine (Malden, Mass.) 2011; 12(6):854–63.

6. Hidayat L, Patel S, Veltri K. Active-learning implementation in an advanced elective course on infectious diseases. American journal of pharmaceutical education 2012; 76(5):87.

7. Gerdhem P. Osteoporosis and fragility fractures: Vertebral fractures. Best practice & research. Clinical rheumatology 2013; 27(6):743–55.

8. Leslie WD, Morin SN. Osteoporosis epidemiology 2013: implications for diagnosis, risk assessment, and treatment. Current opinion in rheumatology 2014; 26(4):440–6.

9. Raisz LG. Pathogenesis of osteoporosis: concepts, conflicts, and prospects. The Journal of clinical investigation 2005; 115(12):3318–25.

10. Sadat-Ali M, Al-Omran A, Al-Bakr W, Azam MQ, Am Tantawy, Al-Othman A. Established Osteoporosis and Gaps in the Management: Review from a Teaching hospital. Annals of medical and health sciences research 2014; 4(2):198–201.

11. Taxel P, Kenny A. Differential diagnosis and secondary causes of osteoporosis. Clinical cornerstone 2000; 2(6):11–21.

12. Davis P, Andrews E, Donen N, Fitzgerald A, Hughes S, Juby A et al. Case studies in osteoporosis: a problem based learning intervention for family physicians. The Journal of rheumatology 1999; 26(11):2418–22.

13. Ghosh S. Combination of didactic lectures and case-oriented problem-solving tutorials toward better learning: perceptions of students from a conventional medical curriculum. Advances in physiology education 2007; 31(2):193–7.

14. Jamkar A, Yemul V, Singh G. Integrated teaching programme with student-centred case-based learning. Medical education 2006; 40(5):466–7.

15. Mishra K, Snow-Lisy DC, Ross J, Goldfarb DA, Goldman H, Campbell SC. Evaluation of a case-based urology learning program. Urology 2013; 82(6):1207–10.

16. Gao X, Luo S, Mu D, Xiong Y, Guanjian L, Wan C. Effects of problem-based learning in paediatric education in China: A meta-analysis. Journal of evidence-based medicine 2016.

17. Zhou J, Zhou S, Huang C, Xu R, Zhang Z, Zeng S et al. Effectiveness of problem-based learning in Chinese pharmacy education: a meta-analysis. BMC medical education 2016; 16:23.

18. Ding X, Zhao L, Chu H, Tong N, Ni C, Hu Z et al. Assessing the effectiveness of problem-based learning of preventive medicine education in China. Scientific reports 2014; 4:5126.

19. Galvao TF, Silva MT, Neiva CS, Ribeiro LM, Pereira MG. Problem-based learning in pharmaceutical education: a systematic review and meta-analysis. TheScientificWorldJournal 2014; 2014:578382.

20. Preeti B, Ashish A, Shriram G. Problem Based Learning (PBL) - An Effective Approach to Improve Learning Outcomes in Medical Teaching. Journal of clinical and diagnostic research : JCDR 2013; 7(12):2896–7.

21. Prosser M, Sze D. Problem-based learning: student learning experiences and outcomes. Clinical linguistics & phonetics 2014; 28(1-2):131–42.

Table and Graph description

Table 1. Elective Osteoporosis Course I test: Comparison of points acquired by study group I and control group I

Table 2. Elective Osteoporosis Course II test: Comparison of points acquired by study group II and control group II

Graph 1. Elective Osteoporosis Course I test: Comparison of points acquired by control group I and study group I

Graph 2. Elective Osteoporosis Course II test: Comparison of points acquired by control group II and study group II

Graph 1

Click here to access/download;Figure;Graph 1.docx

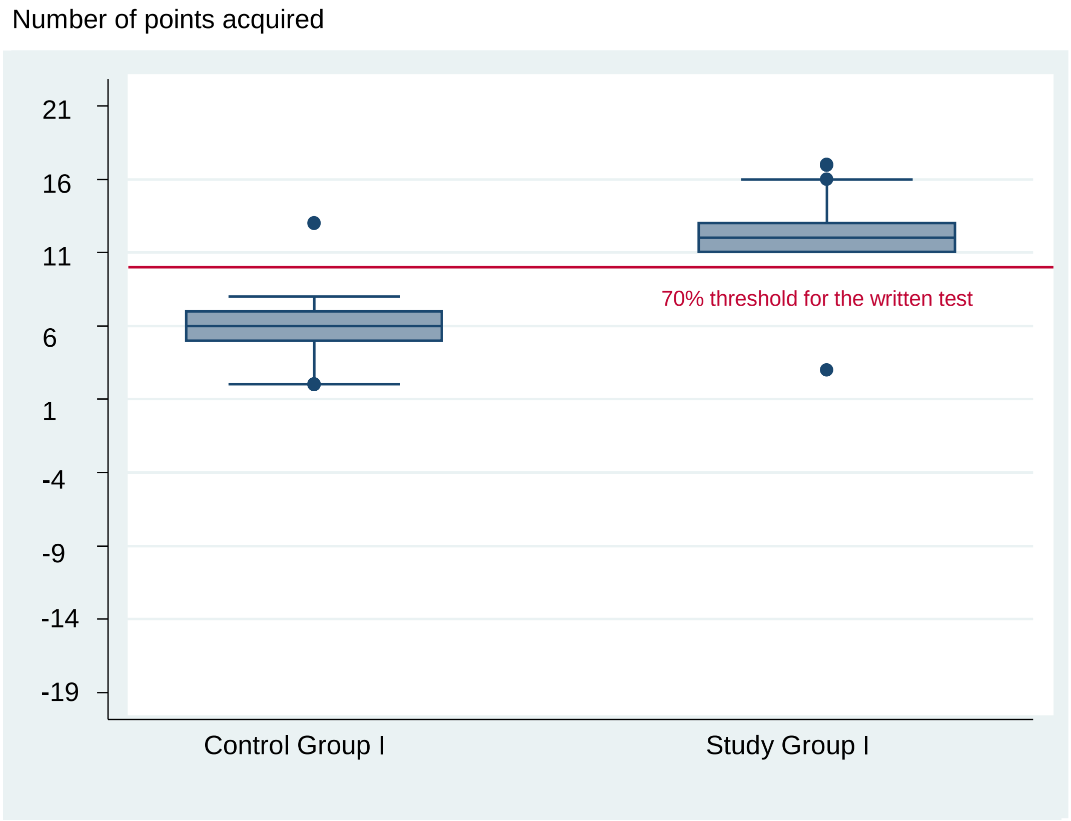

Graph 2

Click here to access/download;Figure;Graph 2.docx

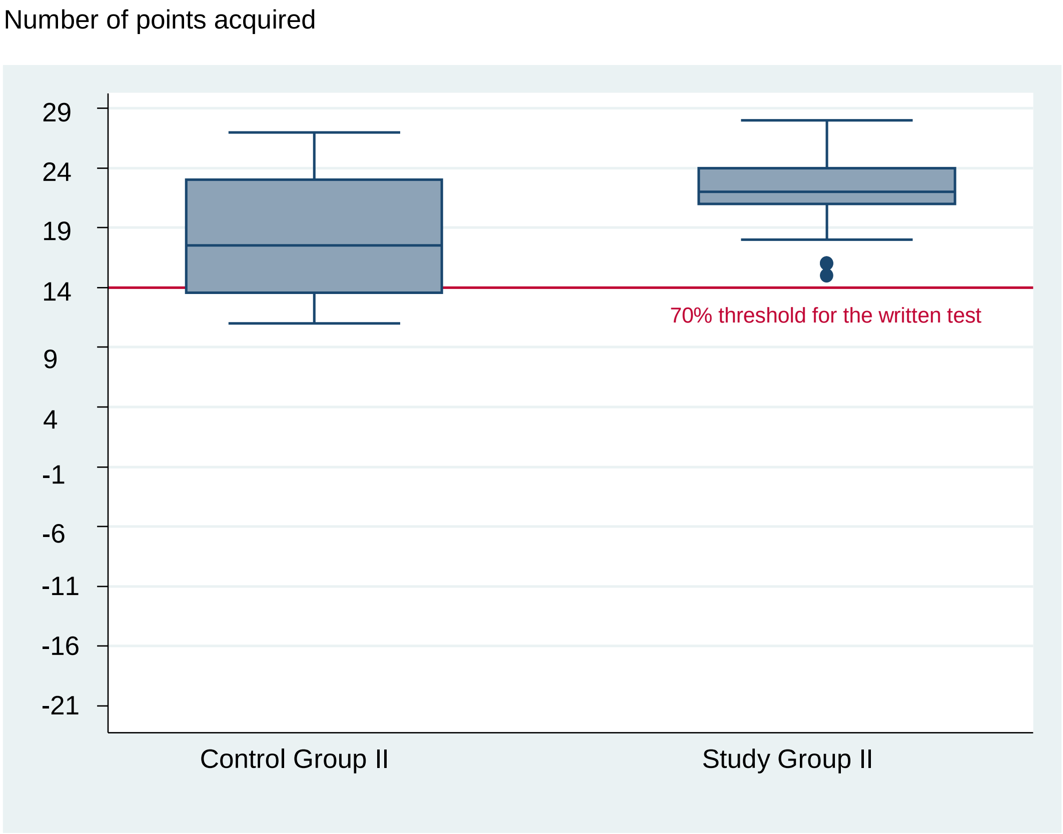

Click here to access/download;Figure;Table 1.docx

Number of points acquired	Number of students Study group I (N^I)	Number of students Control group I (n^I)
2		2
3	1	1
4		3
5		6
6		5
7		6
8		1
9		
10		
11	11	
12	2	
13	5	1
14	1	
15	1	
16	1	
17	3	
Total number of students	25	25

Click here to access/download;Figure;Table 2.docx

Number of points acquired	Number of students Study group II (N^{II})	Number of students Control group II (n^{II})
11		2
12		2
13		2
14		1
15	1	2
16	2	1
17		2
18	1	2
19	2	
20		1
21	5	
22	7	2
23	2	3
24	4	
25		1
26	1	2
27		1
28	2	
Total number of students	27	24
