# Supplementary material for: An osteoporosis course as a separate component of problem-based learning
Source: PLoS One. 2025 Nov 14;20(11):e0336915. doi: 10.1371/journal.pone.0336915 (PMC12617839; doi:10.1371/journal.pone.0336915)
Supplement: Attachment — Submitted filename: The Response to Reviewers.docx [file pone.0336915.s007.docx]

**RESPONSE TO ACADEMIC EDITOR AND REVIEWERS**

Dear Dr. Muhammad Abbas Abid and Reviewers,

Thank you very much for your thoughtful and constructive feedback on our manuscript titled „An Osteoporosis course as a Separate Component of Problem-Based Learning“

We appreciate the time and effort invested in reviewing our work and value the insightful comments that helps us improve the quality and clarity of the manuscript. Below we provide detailed responses to each point raised, along with descriptions of the changes made in the revised manuscript. In the revised version of the manuscript, the changes are highlighted in yellow.

**General response to Additional Editor Comments**

In the **Introduction**, we have added a more comprehensive introduction outlining the epidemiology and significance of osteoporosis as an emerging health problem, including recent prevalence data and the burden of disease.

In the **Methods**, subsection **Participants and groups**, we have clarified the definition and characteristics of the Study and Control groups.

In the **Introduction**, we have explained, why the course was elective.

Potential for self-selection bias are mentioned in the **Methods**, specifically in the end of **Participants and group** subsection, and in the **Disscusion**.

All reviewer comments have been addressed in detail below.

**Responses to Reviewers**

**Reviewer #2**

**Comments in the attached PDF:**

We have addressed all comments, as detailed below:

**Elective Course & Biases:** We clarified the meaning of the Elective Course in the ‚Introduction‘. Potential biases are mentioned in the ‚Methods‘ section, specifically in the end of ‚Participants and group‘ subsection, and in the ‚Disscusion‘ section.

- **Abstract:**
  1. Used optional “Elective Osteoporosis Course” to emphasize the elective nature of the course.
  2. Corrected highlighted words.
  3. Added the test scale in the parenthesis.
- **Introduction:** Provided a more detailed rationale for osteoporosis as a health issue and for the creation of the “Elective Osteoporosis Course”.
- **Methods:**
  1. Explained the sequence of self-study e-learning followed by in-class sessions.
  2. Addressed possible selection bias under Study Groups; omitted the word successfully for Study Group I.
  3. Clarified that all Control Group I students completed all 4th-year and 5th-year osteoporosis-related courses.
  4. Detailed the multiple-choice test methodology and passing criteria.
- **Results:**
  1. Moved the test description and scoring from the ‚Results‘ to the ‚Methods‘ section; clarified credit conditions.
  2. Explained clearly which students passed/failed and received/did not receive credit.
  3. Added missing words.
- **Conclusion:** removed “unequivocally proved” and clarified, why Problem-Based Learning likely contributed to better knowledge retention.

**General comments - major points:**

1. **Non-randomized selection and potential bias:**
   We acknowledge that the elective nature of the course introduces a self-selection bias as students with a particular interest or motivation in osteoporosis may be more likely to enroll. This is now explicitly stated in the ‚Methods‘ section, specifically in the end of ‚Participants and group‘ subsection, Unfortunately, randomization was not feasible given the educational context. We have revised the manuscript to emphasize this limitation and discuss its implications for generalizability in the ‚Disscusion‘ section.
2. **Details on student motivation and background environment:**
   For greater clarity, we have newly divided the 'Methods' section into the following subsections: 'Course Structure', 'Assessment', and 'Participants and Groups'. We added following information in the 'Methods' section: The academic context is described in 'Course Structure' subsection. The criteria for passing or failing the course are described in 'Assessment' subsection. The factors influencing student motivation to take the elective course are mentioned in 'Participants and Groups' subsection.
3. **Suggestion to compare two elective courses:**
   While comparing two elective courses with different teaching methods would be ideal, in this study only the osteoporosis elective was available as an additional course. In the ‚Discussion‘ section, we have noted this as a limitation and suggested it as an area for future research.

**Reviewer #3**

**Major concerns:**

1. **Clarification on groups and additional exposure:**
   For greater clarity, we have newly divided the 'Methods' section into the following subsections: 'Course Structure', 'Assessment', and 'Participants and Groups'. We clarified in the 'Participants and groups' subsection, that all students completed the mandatory curriculum on osteoporosis, and the study groups additionally completed the elective course. This was added to the manuscript to highlight that the elective course was supplementary, which may explain the better test performance in study groups. We mentioned that as a limitation of the study in the 'Methods' section, specifically in the end of 'Participants and groups' subsection, and in the 'Discusion'.
2. **Clarification on test scores and negative values:**
   In the 'Assessment' subsection, we described in detail how the multiple-choice final test was designed, including an explanation of why the achieved scores could range from negative to positive values

**Reviewer #4**

**Detailed methodological clarifications requested:**

1. **Details on PBL implementation:**
   For greater clarity, we have newly divided the 'Methods' section into the following subsections: 'Course Structure', 'Assessment', and 'Participants and Groups'. In the 'Course Structure' we better described course design, with detailed information about the PBL implementation, facilitated by using case study learning. In the 'Participants and Groups' section are better described group sizes, and characteristics.
2. **Comparison of elective vs mandatory courses:**
   In the section 'Participants and Groups' we now better clarified these differences and how the elective course complements the mandatory curriculum. Reasons for the better knowledge retention in PBL course are described in the subsection 'Course Structure', and also mentioned in the ‚Discusion‘.
3. **Clarification of group composition:**	The manuscript now explicitly states that, all Study Group completed the mandatory courses in their study years. Additive value of the elective course is mentioned as a limitation study in the 'Methods' section, specifically in the end of 'Participants and groups' subsection, and in the 'Discusion'.
4. **Academic level differences:** are now better described in the 'Participants and groups' subsection, where the comparison of academic level is mentioned too. In the 'Discusion' is it also mentioned.
5. **Self-selection bias acknowledgment:**
   We acknowledge that the elective nature of the course introduces a self-selection bias as students with a particular interest or motivation in osteoporosis may be more likely to enroll. This is now thoroughly discussed in the „Methods“ section, specifically in the end of „Participants and group“ subsection. We have revised the manuscript to emphasize this limitation in the „Disscusion“ section.
6. **Test version comparability:**
   We clarified design of the final multiple-choice test in the 'Assessment' subsection, including the information, that each set of ten multiple-choice questions was designed to be equivalent in content and to maintain a numerically balanced distribution of response option.
7. **Research procedure description:**
   We added detailed description of timing, test administration, and standardization of conditions in the ‚Assessment“ subsection.
8. **Ethics and consent:**
   We obtained ethics committee approval for this educational research and ensured that all participants provided informed verbal consent. This is now clearly stated in the „Ethics Statement“ section.
9. **Statistical analysis details:**
   In addition to the regression models, Bayes’ theorem was utilized to provide a more comprehensive statistical perspective

**Reviewer #5**

**1. Major concerns:**

For greater clarity, we have newly divided the 'Methods' section into the following subsections: 'Course Structure', 'Assessment', and 'Participants and Groups'. In the 'Participants and Groups' subsection, we clarified that the study groups completed both mandatory and elective courses, while controls completed only mandatory. We explained that all students in Osteoporosis Course II had previously completed Course I, while 2 students were newly included. We mentioned the limitations connected with this topic in the 'Discussion'.

**2. Other Issues:**

1. Study goal:
   The hypothesis and primary outcomes (knowledge gain measured by test scores) are now explicitly stated in the 'Introduction' and 'Methods'.
2. Methods:
   A) We added the 'Participants and Groups' subsection.
   B) Instead of a flowchart, we provided a more detailed characterization of the study groups in the 'Participants and Groups' subsection..
   C) Validity evidence of test tool:
      In the 'Assessment' subsection we described the nature of the test and who developed and validated it.
3. Results and Discusion
   A) Implication of the study are now better mentioned in the 'Conslusion'.
   B) In the section 'Discussion', we decribed limitation of our study.

We hope that these revisions adequately address all concerns and improve the manuscript’s clarity, rigor, and overall quality. We thank the editor and reviewers again for their valuable feedback and look forward to your further consideration.

Sincerely,

On behalf of all authors

Iva Hoffmanová, MD, PhD

Second Faculty of Medicine, Charles University

Prague, Czech Republic
